# Polarization and Trap Characteristics Modification of Oil-Impregnated Paper Insulation by TiO_2_ Nanoparticles

**DOI:** 10.3390/nano9020174

**Published:** 2019-01-31

**Authors:** Meng Huang, Yupeng Ying, Bingliang Shan, Yuzhen Lv, Chengrong Li

**Affiliations:** 1 Beijing Key Laboratory of High Voltage & EMC, North China Electric Power University, Beijing 102206, China; 18811360731@163.com (Y.Y.); shanbingliang521@163.com (B.S.); lcr@ncepu.edu.cn (C.L.); 2 State Key Laboratory of Alternate Electrical Power System with Renewable Energy Sources, North China Electric Power University, Beijing 102206, China; 3 School of Energy, Power and Mechanical Engineering, North China Electric Power University, Beijing 102206, China; yzlv@ncepu.edu.cn

**Keywords:** trap characteristics, TiO_2_ nanoparticles, oil-impregnated paper, interface polarization, hopping

## Abstract

Polarization and traps determine the electrical property of oil-paper insulation, but most attention has been paid to the modification of insulating oil with nanoparticles, so there are is little research about oil-impregnated paper, and the origin for performance variation is not understood yet. In this paper, spherical nanoscale titanium dioxide was prepared by the hydrolysis method and nanofluid-impregnated paper (NP) was fabricated through oil-impregnation. The frequency domain spectrum was measured for polarization analysis, and both thermally stimulated depolarization current (TSDC) and isothermal surface potential decay (ISPD) methods were used to reveal trap parameters. Results show that NP’s low frequency permittivity is much larger, and another peak appears in the spectrum even though the content of nanoparticles is very low. With the addition of TiO_2_ nanoparticles, TSDC’s amplitude and peak temperature increase, and the trap energy becomes shallower. TiO_2_ nanoparticles’ strong polarization and high activation energy contribute to NP’s larger interface polarization intensity and activation energy. Furthermore, because of oxygen vacancies, TiO_2_ nanoparticles offer a transfer site for holes and electrons to escape from deep traps; thus, the trap energy is greatly reduced.

## 1. Introduction

Owing to its excellent electrical performance, oil-paper insulation has been widely used as the main insulating material in large quantities of equipment, such as transformers and cables. However, the development of the electric industry demands miniaturization, large-capacities, and high-reliability for equipment, which challenges the electrical property of conventional oil-paper insulation. The appearance of nanotechnology offers a novel solution to improve oil-paper insulation’s electrical strength by adding nanoparticles [1].

Oil-paper insulation is a kind of complex material which contains liquid and solid dielectrics, so the modification needs to be handled carefully. Since it was found that the breakdown strength of mineral oil can be increased with Fe_3_O_4_ nanoparticles in 1988 [2], it has attracted more and more attention. Different kinds of nanoparticles have been used for the modification of insulating oil, including conductive, semi-conductive, and insulated ones, and the influence of shapes and sizes have been considered as well [2,3,4,5,6,7]. The electrical properties of nanofluids such as breakdown strength and streamer propagation have indeed been markedly improved, and it has been commonly accepted that this is attributed to the variation of dielectric relaxation and trap energy caused by nanoparticles [8,9].

As for the solid component, namely paper or pressboard, it has to be impregnated with oil in application; therefore, its property improvement is usually focused on the modification of oil-impregnated paper or pressboard (both named OP), but there is little research compared to that of oil. Paper is made by pressing fiber together. There are many small pores in it, and therefore the nanoparticles can be made to be absorbed on the surface of fiber before papermaking [10] or transported through the pores and deposited during oil-impregnation [3]. With the modification of nanoparticles, the partial discharge, surface discharge, and breakdown performance of OP can be perfected to some degree [10,11,12]. These changes are believed to be strongly linked to dielectric polarization and space charge suppression [11,12]. Permittivity of modified OP depends on nanoparticles’ type, which can be either decreased—by SiO_2_ nanoparticles, for instance—or increased—by TiO_2_ nanoparticles, for instance [10,13]. Charge accumulation behavior of AlN-modified OP is found to be affected by nanoparticle concentration because of the competition between the interfacial and the agglomeration effect, and, furthermore, the property may instead suffer under some conditions [14]. The electric field distribution within OP is closely related to its electrical property, including breakdown and partial discharge, which is determined by permittivity and space charge [12,15,16]. Nevertheless, the underlying reason for their variations in modified OP has not yet been clearly demonstrated; therefore, the influence of nanoparticles on polarization and trap characteristics requires consideration.

In this paper, anatase-TiO_2_ nanoparticles were used for modification through the oil-impregnation method, and their effect on polarization and trap distribution was analyzed to reveal the behind mechanism. In addition to an interface increase, TiO_2_ nanoparticles’ large permittivity leads to a strong interface polarization of the nanofluid-impregnated paper or pressboard (both named NP) with higher activation energy. The oxygen vacancies from TiO_2_ nanoparticles help the trapped charge to dissipate and will thus weaken the electric field distribution.

## 2. Materials and Methods

**Figure 1:**
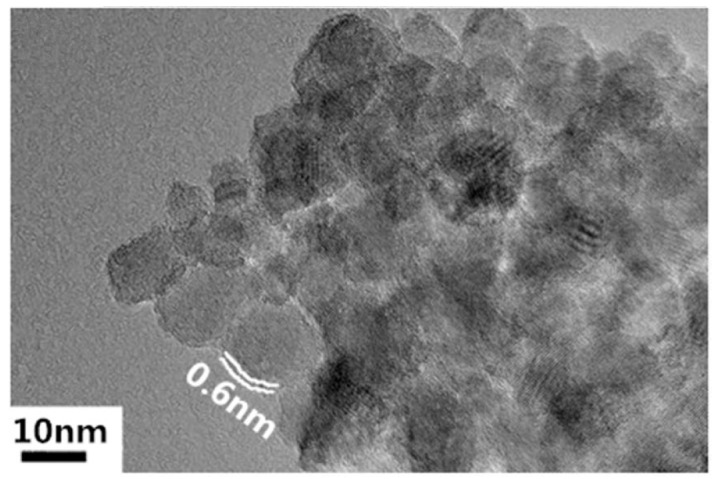
Transmission electron microscopy (TEM) image of TiO_2_ nanoparticles.

Nanoscale titanium dioxide was prepared by the hydrolysis method using four butyl titanate as the titanium source and oleic acid as the surface modifier. Sizes of spherical nanoparticles can be controlled by adjusting the reaction environment, such as the amount of oleic acid and temperature [3]. Under proper conditions, 10 nm TiO_2_ nanoparticles—the transmission electron microscopy (TEM) image of which is shown in Figure 1—were fabricated, and they were then dispersed into the strictly filtered mineral transformer oil (Karamay 25#) which fulfilled the quota by CIGRE working group 12.17.10 through the ultrasonic method [3]. The concentration of nanoparticles in the nanofluid was 0.075 vol%. Before impregnation, the 1 mm thick B3.1 type pressboard from Weidmann Company was also carefully handled. It was first cut into pieces with sizes of 85 mm × 60 mm. Then, they were put into a ventilated 105 °C oven for 48 h to remove the moisture. After that, these pressboard pieces were put into a 1 kPa/85 °C vacuum for 48 h. The nanofluid and pure transformer oil were treated in the same way. After that, dried pressboards were put into either pure oil or nanofluid in a 1 kPa/80 °C vacuum for 48 h for oil-impregnation. Through these pretreatments, the moisture content of both OP and NP did not exceed 0.5%.

**Figure 2:**
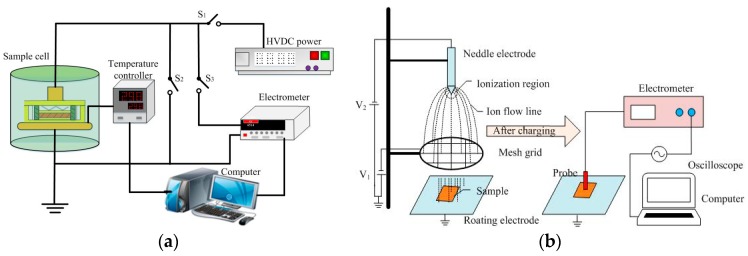
Schematic diagrams of: (**a**) The thermally stimulated depolarization current (TSDC) method and (**b**) the isothermal surface potential decay (ISPD) method.

Trap distribution characteristics of NP and OP were measured by both thermally stimulated depolarization current (TSDC) and isothermal surface potential decay (ISPD) methods. The schematic diagrams are demonstrated in Figure 2. Details of TSDC measurement can be found in our previous paper [17]. In regard to the ISPD method, it consisted of two processes—namely surface charging and surface potential measurement. During the surface charging process, a needle electrode connected to high voltage was used for ion generation of either sign through corona discharge. The wire mesh grid was used to generate a uniform field between the mesh grid and ground electrode for a better charge [18]. The needle electrode was applied with ±6 kV and the mesh grid was connected to ±3 kV DC voltage. Immediately after the corona discharge, the sample was quickly transferred to below the Kelvin probe for isothermal surface potential measurement through the rotating grounded electrode. The distance between the sample and non-contact probe (Trek P0865) was fixed at 2 mm. At the beginning of the ISPD experiment, the ground potential calibration was carried out.

An inductively coupled plasma optical emission spectrometer (Agilent 5110 ICP-OES) was used to detect the content of titanium element in both OP and NP. The frequency domain spectrum of their permittivity was measured by an IDAX 300 Insulation Diagnostic Analyzer at ambient temperature, with the voltage being fixed to 140 V.

## 3. Results

### 3.1. Content of Titanium Element

**Figure 3:**
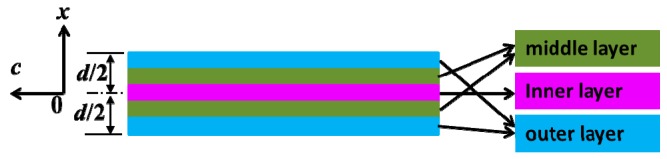
Schematic diagrams positions and different layers in impregnated pressboards.

The impregnated pressboards can be divided into five layers for measurement of the content of titanium element, as shown in Figure 3. Results for both NP and OP are listed in Table 1. Those of NP which had been scoured with pure oil through stirring for different times are included in Table 1 as well.

Though there was no titanium element in OP, there was such a high content of titanium element in NP due to the diffusion of TiO_2_ nanoparticles. The content reached as high as 160.0 ppm in the outer layer, and it decreased gradually from the outer surface to the inner layer. Scoured with pure oil, the content of titanium element in NP decreased obviously, but the deeper from the outer surface, the smaller the content variation. For example, content of titanium element of the outer layer reduced from 160.0 ppm to 10.2 ppm when it had been washed for 168 h, while it just changed from 44.5 ppm to 31.2 ppm for the inner layer. If the time duration of scouring had extended to 336 h, the content varied much more apparently. This meant that there was no direct bond formation between nanoparticles and cellulose in NP, and they just stayed in the small pores within the pressboards, which can diffuse through NP along with the movement of oil.

### 3.2. Frequency Domain Spectrum

**Figure 4:**
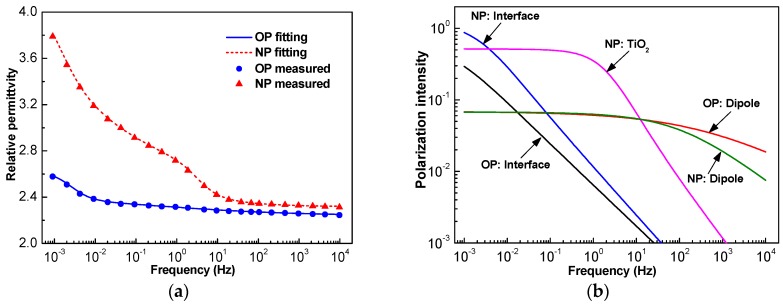
Frequency domain spectrum of oil-impregnated paper (OP) and nanofluid-impregnated paper (NP): (**a**) measured permittivity and its fitting curve; (**b**) their composition of different dielectric relaxation processes.

The frequency domain spectrum of both OP and NP (170 μm paper was used here) permittivity is shown in Figure 4a. In the high frequency region, the permittivity of both OP and NP kept almost unchanged with the variation of frequency. However, in the low frequency region, the permittivity increased with the decrease of frequency. The permittivity and variation trends of NP were larger than that of OP, especially when the frequency was lower than 10 Hz.

For a dielectric consisting of multiple Cole-Cole relaxation processes, its frequency dependent permittivity can be described as [19]:

(1) ε=ε∞+Re(∑i=1nΔεi1+(jωτi)αi)

where *ε*_∞_ is the optical frequency permittivity, *n* is the quantity of different relaxation processes, and *τ_i_*, *α_i,_* and ∆*ε_i_* are the relaxation time, distribution function, and relaxation intensity of the *i*-th relaxation process, respectively. It has been proved that there are generally two processes in OP [20], and it can be seen that there was one more peak around 1 Hz for NP. Hence, there would be one more relaxation process in NP. Through least square fitting, the parameters of each relaxation process could be obtained and are illustrated in Table 2, and the corresponding curves of fitted frequency dependent permittivity and each process are presented in Figure 4 as well.

The addition of TiO_2_ nanoparticles caused the interface polarization intensity to increase dramatically. The relaxation time of the dipole polarization also distinctly increased, but its polarization intensity reduced a little. The third relaxation process of NP is very clear at a glance in Table 2 and Figure 4b.

### 3.3. Thermally Stimulated Depolarization Current

**Figure 5:**
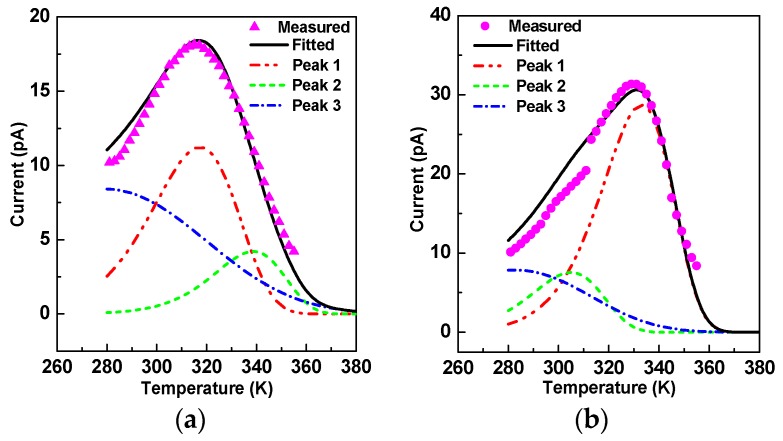
Measured TSDC curves and their fitted and separated peaks: (**a**) peaks of OP; (**b**) peaks of NP.

The current peak appeared at the temperature ranges from 280 K–380 K and reflects the interface polarization in oil-impregnated paper insulation. It can be described as follows [21]:

(2) $$I\left(T\right)=\frac{p_{0}}{\tau_{0}}\exp\left[-\frac{E_{a}}{kT}-\frac{1}{\beta \tau_{0}}\int_{T_{0}}^{T}\exp\left(-\frac{E_{a}}{kT}\right)dT\right]$$

where *p*_0_ is polarization intensity, *τ*_0_ is relaxation time, *E*_a_ is activation energy, *T* is temperature, *T*_0_ is initial temperature, *k* is Boltzmann constant, and *β* is temperature arising rate, which was 2 K/min in this paper. The measured results are displayed in Figure 5, and the peak point—separately appearing at 315 K for OP and 330 K for NP—belonged to the interface polarization region. However, the peak widths were a little wider; if we calculated the activation energy using *E*_a_ = 2.47${kT}_{m}^{2}$/Δ*T* (where Δ*T* is half-peak width and *T*_m_ is peak point temperature), it would be smaller [11,21]. Therefore, the peaks were fitted and separated with the consideration that the peak width was just affected by the neighboring peaks. After peak fitting, there were three peaks for both OP and NP at the three same peak temperatures; each of them had an obvious main peak, which was labeled as peak 1 in Figure 5. Just the main peaks were analyzed by curve fitting to equation (2), and it was determined that NP’s activation energy was 0.64 eV and OP’s was 0.48 eV.

### 3.4. Isothermal Surface Potential Decay

**Figure 6:**
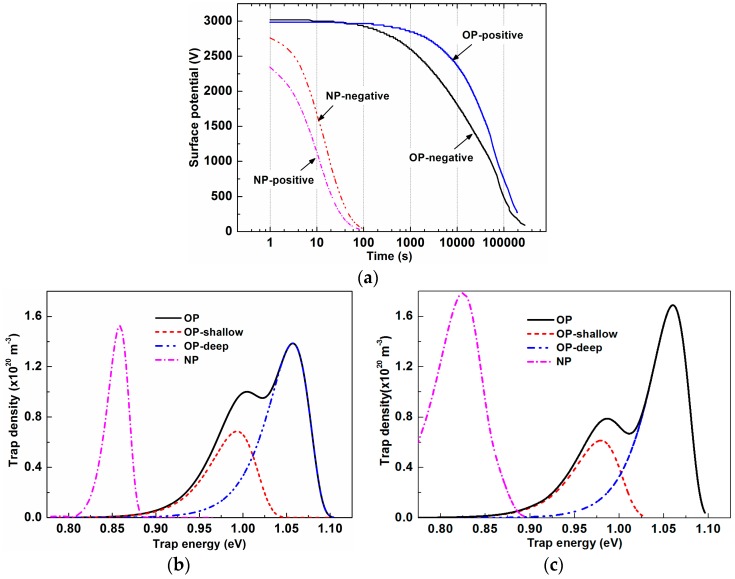
Measured ISPD curves and the corresponding calculated energy distribution: (**a**) Time dependent surface potential; (**b**) electron trap energy distribution; (**c**) hole trap energy distribution.

The measured ISPD curves under both negative and positive coronae are given in Figure 6a with the *x*-axis on a logarithmic scale. The surface potential of NP decayed very quickly, and it decreased to approximately 0 V within 100 seconds no matter whether it was negatively or positively charged. In regard to OP, its surface potential decayed much more slowly, and it was still larger than 500 V after 10^5^ seconds. However, the negative potential varied a little faster than the positive one. Based on the ISPD curves, trap energy and density can be obtained by using [22]:

(3) $$E_{t}=kT\ln\left(\nu t\right)$$

(4) $$N\left(E_{t}\right)=\frac{\varepsilon_{0}\varepsilon_{r}}{elL}t\frac{\partial \phi_{s}\left(t\right)}{\partial t}$$

where *ε*_0_ is vacuum permittivity, *ε*_r_ is the sample’s relative permittivity, *l* is penetration depth (fixed as 1 μm), *L* is the thickness of the sample, *ϕ*_s_ is the surface potential, *e* is electron charge, *t* is time, and *ν* is the attempt-to-escape frequency. Calculated trap characteristics are shown in Figure 6b,c, and the relevant parameters are listed in Table 3.

Both electron and hole traps of OP consisted of two peaks that can be divided into a shallow and deep peaks through peak fitting. The center trap energy of deep traps was 0.08 eV higher than that of shallow traps, regardless of trap polarity. For shallow traps, peak density of the holes was 14.5% smaller than that of electrons and 20% larger than electron traps’ peak density for deep ones. The peak density of deep traps was about twice that of shallow traps no matter what polarity it was. TiO_2_ nanoparticles made a significant difference on trap distributions. There was only one peak for NP’s electron traps as well as hole traps. Though the peak width of the holes was a little wider than that of electrons, NP’s peak density of electrons was similar to that of hole traps, and they were close to those of OP’s sum of deep and shallow traps. However, NP’s trap energies were much lower than those of OP’s shallow traps, not to mention those of deep traps.

## 4. Discussion

As mentioned before, because of the stable chemical property of cellulose, it is difficult to fabricate fiber with nanoparticles surrounded by cellulose like other solid nano-dielectrics, such as LDPE/MgO nanocomposites [23]. Hence, if one wants to improve the electrical property of OP with nanoparticles, there are generally two possible approaches. The first one is to mix nanoparticles with wood pulp and then stir them before paper sheet preparation [10,14]. The nanoparticles are adsorbed on the surface of fibers, which results in a certain content of nanoparticles in pressboard. The other one is just to make pressboard impregnated with nanofluids [11]. Nanoparticles will transport through the pressboard along the movement of nanofluid, and there will be a deposition of them [24]. No matter which way is used, the main mechanism of nanoparticles addition is adsorption (as well as deposition caused by collision), hence the nanoparticles’ distribution is influenced by the size of the small pores in pressboard and the movement of oil [24,25,26]. The NP was made through the latter way in this paper. Therefore, just the deposition with oil flow was analyzed. The typical transport-deposition model can be described as [27]:

(5) $$\frac{\partial c}{\partial t}=D_{L}\frac{\partial^{2}c}{\partial x^{2}}-u\frac{\partial c}{\partial x}-K_{\mathrm{dep}}c$$

(6) $$A\frac{\partial s}{\partial t}=K_{\mathrm{dep}}c,$$

and the initial and boundary conditions can be set as:

(7) $$\left\{ \begin{array}{l} c\left(t=0,x\right)=0\\ c\left(t,x=d/2\right)=c\left(t,x=-d/2\right)=c_{0} \end{array}\right.$$

where *c* is the time- and position-dependent nanoparticle density, *s* is the deposited nanoparticles’ density, *d* is the pressboard’s thickness, *x* is position (as illustrated in Figure 3), *c*_0_ is the nanoparticles’ density in oil, *A* is a constant related to dry pressboard’s density and porosity, *D*_L_ is the longitudinal diffusion coefficient, *u* is seepage velocity, and *K*_dep_ is the deposition coefficient. By solving Equations (5) and (6), nanoparticles’ distribution can be obtained as:

(8) $$\begin{array}{ll} c\left(x,t\right) & =\frac{c_{0}}{2}\exp\left[\frac{u\left(0.5d+x\right)}{2D_{L}}\left(1-\phi \right)\right]\mathrm{erfc}\left(\frac{0.5d+x-ut\phi }{\sqrt{4D_{L}t}}\right)+\frac{c_{0}}{2}\exp\left[\frac{u\left(0.5d+x\right)}{2D_{L}}\left(1+\phi \right)\right]\mathrm{erfc}\left(\frac{0.5d+x+ut\phi }{\sqrt{4D_{L}t}}\right)\\ & +\frac{c_{0}}{2}\exp\left[\frac{u\left(0.5d-x\right)}{2D_{L}}\left(1-\phi \right)\right]\mathrm{erfc}\left(\frac{0.5d-x-ut\phi }{\sqrt{4D_{L}t}}\right)+\frac{c_{0}}{2}\exp\left[\frac{u\left(0.5d-x\right)}{2D_{L}}\left(1+\phi \right)\right]\mathrm{erfc}\left(\frac{0.5d-x+ut\phi }{\sqrt{4D_{L}t}}\right) \end{array}$$

where erfc(*x*) is the residual error function, $\phi =\sqrt{1+\frac{4K_{\mathrm{dep}}D_{L}}{u^{2}}}$.

**Figure 7:**
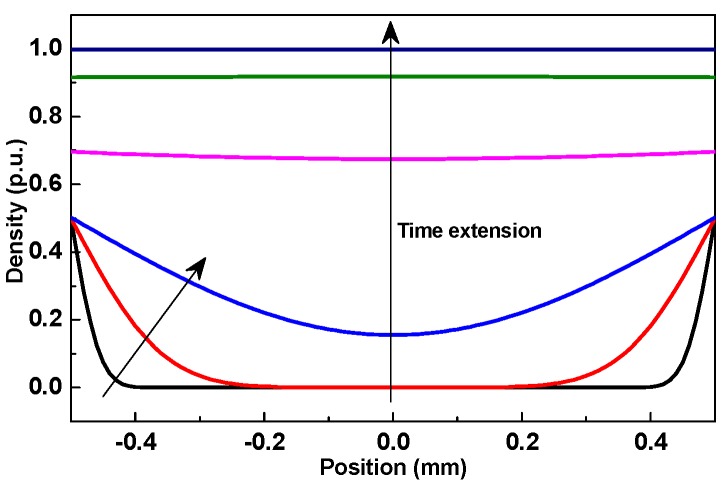
Normalized time and position dependent nanoparticles’ density within NP.

Longitudinal diffusion and deposition coefficients are greatly affected by particle size and seepage velocity [25], but these parameters about TiO_2_ nanoparticles in NP are absent at present. Choosing *u* = 0.1 cm/s, *D*_L_ = 0.06 cm^2^/s, and *K*_dep_ = 0.006 s^−1^ from other particles to demonstrate the time dependent variation, we got the normalized nanoparticles’ distribution within NP, as shown in Figure 7. Nanoparticles move from the outside to the inside, and the density gradually decreases from outer surface to inner layer at the beginning, which agrees with the result in Table 1. When the time duration is long enough, a uniform distribution of nanoparticles should appear; however, this actually cannot happen, as there are surface and blocking sieving besides deposition, and the subsequent deposition will be influenced [26]. For lack of strong bending force [28], when NP was scoured with pure oil, the TiO_2_ nanoparticles’ concentration gradient and fluid’s flow made them move from inside to outside, and it was increasingly easier for them to move from the inner layer to the outer surface. Thus, the decrease of the nanoparticles’ density increases with washing duration and depth from inner layer. The time- and position-dependent variation of the nanoparticles’ density means that the nanoparticles cannot stably remain in NP. Therefore, the nanoparticles’ transport and the process of making a stable distribution need further study because nanoparticle density makes a great difference on the electrical property [10,14].

We used anatase TiO_2_ nanoparticles, the relative permittivity of which can be as high as 48 and dramatically increase in the low frequency region. Hence, there is a peak in the frequency domain spectrum reflecting the polarization of TiO_2_ nanoparticles, and its relaxation time is 0.08 s. Besides, TiO_2_ nanoparticles are semi-conductive. Specifically, their conductivity is much larger than that of OP. There is therefore a huge difference on permittivity and conductivity between OP and TiO_2_ nanoparticles, and though the concentration of TiO_2_ was just 0.075 vol%, interface polarization was very great. It has been increased approximately three times, as shown in Figure 3b. The TSDC peak at about 315 K corresponds to the interface polarization between oil and fiber [21]. Nevertheless, it has appeared as peak 1 in Figure 5a for OP and peak 2 in Figure 5b for NP. The interface polarization of NP between oil and fiber decreased a little because nanoparticles are deposited on the surface of the fiber. On the other hand, there was a much higher peak at 330 K for NP, and its activation energy was about 0.64 eV. By testing the temperature-dependent permittivity of 18 nm anatase TiO_2_ nanoparticles, it was found that a peak appears at about 340 K when the frequency is fixed to 96 kHz [29]. According to the absolute-rate theory by Eyring, relaxation time depends on activation energy and temperature:

(9) $$\frac{1}{\tau }=\frac{kT}{h}\exp\left(-\frac{E_{a}}{RT}\right)$$

where *h* is Planck’s constant—*h* = 6.63 × 10^−34^ J∙s—and *R* is gas constant—*R* = 8.314 J/(mol∙K). Then, the activation energy for TiO_2_ nanoparticles’ polarization can be obtained as 0.53 eV, which is a little lower than the activation energy measured by TSDC. This is because the interface polarization can actually happen between TiO_2_ nanoparticles with either fiber or oil. It is induced by their permittivity differences. As a result, its relaxation time is longer and the activation energy is higher. All of those can contribute to the TSDC, and they are so closely related [30] that it is beyond the resolution capability of TSDC. Similarly, the calculated activation energy was found to be 0.67 eV using the relaxation time in Table 3.

On account of this strong interface polarization, the orientation of some dipoles is restricted. As a result, the dipole polarization of NP decreases a little, but its relaxation time increases a lot. Dipole polarization is very fast, and its TSDC peak point temperature is 198 K [21], so part of peak 3 can be seen in Figure 5.

**Figure 8:**
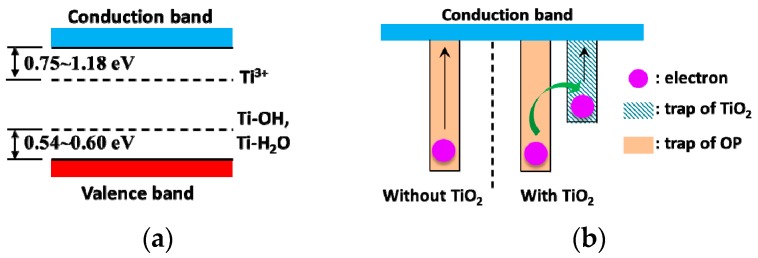
Schematic diagram of electric band structure and electron movement: (**a**) Band structure of anatase TiO_2_ with oxygen vacancy; (**b**) schematic diagram illustrating process of electron escape.

Generally, there inevitably are many oxygen vacancies in anatase-TiO_2_ nanoparticles. These oxygen vacancies can cause electron localization at original position, which will directly affect the electron structure of TiO_2_. Unpaired electrons or Ti^3+^ can be generated consequently, and they act as shallow donor sites 0.75~1.18 eV below the conduction band [31,32]. Meanwhile, though the water content is very limited, there are a certain number of water molecules in NP. H_2_O molecules can be adsorbed on TiO_2_ nanoparticles’ surfaces, and some of them can then be dissociated through the forming of bridged hydroxyl groups [33]. Ti-OH and Ti-H_2_O act as a surface states 0.60 eV and 0.54 eV above the valence band separately [34]. Therefore, the band structure of TiO_2_ nanoparticles can be illustrated as Figure 8a. Together with Figure 6, it can be seen that the trap energy of TiO_2_ is much lower than that of OP. From Equation (3), we can get the time for an electron to escape from a trap:

(10) $$t=\frac{1}{\nu }\exp\left(\frac{E_{t}}{kT}\right)$$

**Figure 9:**
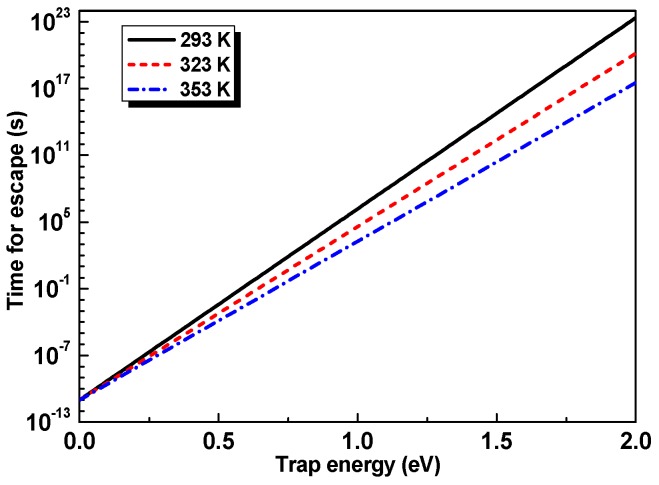
Time for an electron to escape from a trap with different energies at different temperatures.

The corresponding trap energy-dependent time is displayed in Figure 9. It can be seen that even though the trap energy increases a little, the time for escape is many times longer. It takes about 80 s for an electron to escape from a trap in TiO_2_ nanoparticles. However, it takes about 1.5 × 10^6^ s for an electron to escape from a trap of 1 eV in OP. After the addition of TiO_2_ nanoparticles—as the trap energy difference between OP and TiO_2_ nanoparticles is about 0.4 eV—an electron can move between traps through tunneling or jumping mechanisms [35]. This is awfully fast and only needs 7.5 × 10^−5^ s, which can be ignored with respect to the time for escape from a trap in either OP or TiO_2_ nanoparticles. ISPD results verify that the time is mostly spent on escaping from traps in TiO_2_ nanoparticles for NP. This means that the electron first transfers from traps in OP to those in TiO_2_ nanoparticles before escaping in NP (and so does the hole), as illustrated in Figure 8b. As a result, the measured trap energy becomes shallower.

## 5. Conclusions

The addition of TiO_2_ nanoparticles restricts the dipole polarization but enhances the interface polarization. Since TiO_2_ nanoparticles possess a larger permittivity and activation energy, TiO_2_ nanoparticles’ polarization makes a significant contribution to a new peak of NP’s permittivity spectrum, and the activation energy for interface polarization is increased.

Due to oxygen vacancies, TiO_2_ nanoparticles offer a springboard for electron and hole transport. They jump into the traps of TiO_2_ nanoparticles at first, and then they quickly escape from these traps. Hence, the time for escape is greatly shortened, thereby introducing shallow traps.

Because there is a lack of strong bending force between TiO_2_ nanoparticles and fiber, movement of TiO_2_ nanoparticles in OP is possible but complex, and their distribution is not stable or controllable. This requires further study.

## Figures and Tables

**Table 1:** Content of titanium element in different layers in impregnated pressboards (Unit: ppm).

Sample	Inner Layer	Middle Layer	Outer Layer
OP	0.0	0.0	0.0
NP	44.5	106.0	160.0
NP scoured for 168 h	31.2	85.6	10.2
NP scoured for 336 h	16.5	58.9	1.17

**Table 2:** Estimated multiple relaxation Cole-Cole model parameters of OP and NP.

Sample	*ε*_∞_	Interface Polarization			Dipole Polarization			TiO_2_ Polarization		
		∆*ε*_1_	*τ*_1_	*α*_1_	∆*ε*_2_	*τ*_2_	*α*_2_	∆*ε*_3_	*τ*_3_	*α*_3_
OP	2.23	0.71	262.3	0.57	0.069	3.1 × 10^−4^	0.31	—	—	—
NP	2.32	1.25	63.49	0.67	0.068	9.9 × 10^−4^	0.45	0.51	0.084	0.79

**Table 3:** Estimated traps parameters of OP and NP.

Parameter	NP		OP			
			Shallow Traps		Deep Traps	
	Electron	Hole	Electron	Hole	Electron	Hole
Trap energy (eV)	0.86	0.83	0.98	0.98	1.06	1.06
Trap density (10^20^ m^−3^)	2.23	2.42	0.71	0.62	1.40	1.69
